# Distinguishing Benign and Malignant Thyroid Nodules and Identifying Lymph Node Metastasis in Papillary Thyroid Cancer by Plasma *N*-Glycomics

**DOI:** 10.3389/fendo.2021.692910

**Published:** 2021-06-25

**Authors:** Zejian Zhang, Karli R. Reiding, Jianqiang Wu, Zepeng Li, Xiequn Xu

**Affiliations:** ^1^ Department of Medical Research Center, State Key Laboratory of Complex Severe and Rare Diseases, Peking Union Medical College Hospital, Chinese Academy of Medical Sciences and Peking Union Medical College, Beijing, China; ^2^ Biomolecular Mass Spectrometry and Proteomics, Bijvoet Center for Biomolecular Research and Utrecht Institute for Pharmaceutical Sciences, University of Utrecht, Utrecht, Netherlands; ^3^ Netherlands Proteomics Center, Utrecht, Netherlands; ^4^ Department of Clinical Laboratory, Peking Union Medical College Hospital, Chinese Academy of Medical Sciences and Peking Union Medical College, Beijing, China; ^5^ Department of General Surgery, Peking Union Medical College Hospital, Chinese Academy of Medical Sciences and Peking Union Medical College, Beijing, China

**Keywords:** plasma protein *N*-glycome, sialylation, fucosylation, biomarker, mass spectrometry, lymph node metastasis

## Abstract

**Background:**

Biomarkers are needed for patient stratification between benign thyroid nodules (BTN) and thyroid cancer (TC) and identifying metastasis in TC. Though plasma *N*-glycome profiling has shown potential in the discovery of biomarkers and can provide new insight into the mechanisms involved, little is known about it in TC and BTN. Besides, several studies have indicated associations between abnormal glycosylation and TC. Here, we aimed to explore plasma protein *N*-glycome of a TC cohort with regard to their applicability to serve as biomarkers.

**Methods:**

Plasma protein *N*-glycomes of TC, BTN, and matched healthy controls (HC) were obtained using a robust quantitative strategy based on MALDI-TOF MS and included linkage-specific sialylation information.

**Results:**

Plasma *N*-glycans were found to differ between BTN, TC, and HC in main glycosylation features, namely complexity, galactosylation, fucosylation, and sialylation. Four altered glycan traits, which were consecutively decreased in BTN and TC, and classification models based on them showed high potential as biomarkers for discrimination between BTN and TC (“moderately accurate” to “accurate”). Additionally, strong associations were found between plasma *N*-glycans and lymph node metastasis in TC, which added the accuracy of predicting metastasis before surgery to the existing method.

**Conclusions:**

We comprehensively evaluated the plasma *N*-glycomic changes in patients with TC or BTN for the first time. We determined several *N*-glycan biomarkers, some of them have potential in the differential diagnosis of TC, and the others can help to stratify TC patients to low or high risk of lymph node metastasis. The findings enhanced the understanding of TC.

## Introduction

Thyroid nodules (TN) are the most common thyroid disease and its incidence has been increasing worldwide in recent years. Studies revealed a prevalence of 2 to 6% with neck palpation, 19 to 35% with sensitive imaging devices (such as ultrasound diagnostic systems) and 8 to 65% in autopsy data (1, 2). Although around 90% of TN are benign, in 10% of cases TN predispose to thyroid cancer (TC). For the patients with malignant TN, most of them require timely thyroidectomy or other treatment such as central cervical lymph node resection. In some cases, hemithyroidectomy/active surveillance could be pursued. For the patients with benign thyroid nodules (BTN), a large proportion of them only needs standardized and regular follow-up, except for some special cases (e.g., nodular goiter) requiring surgery (3–5). Therefore, in order to facilitate clinical decision-making, it is tremendously important to preoperatively distinguish between benign and malignant TN. Clinically, the preoperative diagnosis of benign or malignant TN is not always straightforward and lacks a standard test. The routine examination procedures usually rely on a combination of ultrasound and fine-needle aspiration (FNA) cytology. FNA cytology is always chosen to evaluate the malignant risk when the TN are suspected as malignancy by ultrasound (6). Nevertheless, cytological uncertainty is present in 20 to 30% of FNA samples (7), which are classified as indeterminate thyroid nodules (ITN). Most patients with ITN are referred to surgery. Nevertheless, more than half of the ITN are identified as BTN by postsurgical pathology (8). Consequently, more than half of the patients with ITN underwent unnecessary surgeries, which brings psychological burden and an overload of medical expenditure for the patients and results in lifetime thyroxine supplementation. Besides, FNA cytology is an invasive method and patients with ITN have to suffer from both mental and physical trauma. Therefore, more precise and non-invasive molecular methods are urgently needed to preoperatively identify benign or malignant TN. In addition, it’s reported that 30–80% of TC can occur cervical lymph node metastasis, which leads to a 10–42% increase in recurrence rate and a corresponding increase in patient mortality (9). How to indicate whether a patient has lymph node metastatic cancer before surgery is another key problem in the clinic of TC (9, 10). At present, clinicians often need to judge based on personal experience (10). Non-invasive diagnostic biomarkers for stratifying TC patients (low or high risk of metastasis) preoperatively are of great importance to surgical decision-making and reducing the long-term recurrence rate of TC.

Glycosylation is the most prevalent posttranslational modification of proteins that can greatly affect the structural and functional properties of the proteins (11, 12). The modification has effects in many biological processes such as protein secretion, degradation, transport to receptor interaction, and modulation of the immune response (12, 13). Furthermore, it has been reported that glycosylation is involved in the pathophysiology of various major diseases including cancer (14, 15). Protein glycomic signatures can dramatically change due to pathologic conditions (16, 17) and it has been revealed that aberrant glycosylation may be a result of initial carcinogenic transformation (18, 19). In addition, researchers found that altered glycosylation promoted cancer immune suppression and metastasis (20). Investigation of glycosylation profiles in the context of cancers may provide insight into the mechanisms regarding tumor progression and metastasis and help develop novel methods for the detection and prediction of specific cancer types. For the past few years, serological glycomic profiling provides a new approach for the discovery of non-invasive biomarkers. The total plasma protein *N*-glycome has been increasingly reported to have great potential as biomarkers in a multitude of diseases, especially cancer (15, 21–24). Several studies have indicated associations between abnormal glycosylation and TC (25–27), which exemplified a biomarker potential of the altered glycans. Though blood-based biomarker tests may offer a non-invasive and cost-effective way to detect or predict the disease, little is known about the plasma *N*-glycosylation profiles in BTN and TC.

In the present study, we evaluated the plasma *N*-glycome features of three subgroups including malignant and benign TN and HC. As the functions of sialylation depend on the linkage type, the here employed workflow applied linkage-specific sialic acid derivatization with discrimination between α2,3- and α2,6-linked sialic acids on the released *N*-glycans from plasma, followed by matrix-assisted laser desorption/ionization time-of-flight mass spectrometry (MALDI-TOF-MS) analysis and automated data processing (28, 29). We sought to reveal differences in the plasma *N*-glycome of TC, BTN, and HC and discover non-invasive glycan markers for differential diagnosis of malignant and benign TN and preoperatively stratifying TC patients (low or high risk of metastasis), as well as provide insight into the possible involvement of plasma *N*-glycans in the early oncogenic events and metastasis of TC.

## Material and Methods

### Study Population and Sample Collection

Plasma samples obtained from 75 patients diagnosed with TC, 25 patients diagnosed with BTN, and 50 HC were consecutively collected between June 2019 and November 2020 from the Peking Union Medical College Hospital (Beijing, China). The three subgroups were age- and sex-matched as far as possible. HC were defined by medical doctors according to eligibility criteria and they should have no history of systematic diseases, have normal thyroid ultrasound, normal thyroid function, and biochemical parameters. Patients with BTN or TC were diagnosed on the basis of ultrasound and FNA and were confirmed by surgical histopathology. Ultrasound was performed by the same group in the present study. We used the American College of Radiology Thyroid Imaging Reporting and Data System (TI-RADS) for ultrasound features of the nodules. The ultrasound features and more detailed information on the cohort are presented in **Table 1**. All patients with TC were clinically classified as papillary thyroid carcinoma (PTC). We obtained approval from the regional ethics committee of the Peking Union Medical College Hospital and informed written consents from all participants were acquired.

**Table 1:** Clinicopathological characteristics of all participants by subgroup.

	TC	BTN	HC
**N**	75	25	50
**age, mean (range)**	41.85 (24–58)	45.92 (18–67)	40.10 (25–61)
**gender, male (%)**	25 (33.33%)	9 (36.00%)	17 (34.00%)
**without lymph node metastasis, n (%)**	37 (49.33%)	\	\
**with lymph node metastasis, n (%)**	38 (50.67%)	\	\
**pathological types**	PTC	\	\
**size of the nodules (cm)**			
**length (median, IQR)**	1.25 (0.93–1.63)	2.35 (0.70–3.55)	\
**width (median, IQR)**	1.00 (0.80–1.43)	2.10 (0.62–3.35)	\
**height (median, IQR)**	0.80 (0.65–1.08)	1.70 (0.61–2.33)	\
**blurred margins, n (%)**	35 (46.67%)	6 (24.00%)	\
**irregular margins, n (%)**	35 (46.67%)	6 (24.00%)	\
**TI-RADS category, n**			
**2–3**	1	11	**\**
**4**	27	8	**\**
**5**	47	4	**\**

The data for size, margins, and TI-RADS is the ultrasound features. TC, thyroid cancer; BTN, benign thyroid nodules; HC, healthy controls; PTC, papillary thyroid carcinoma; TI-RADS, American College of Radiology Thyroid Imaging Reporting and Data System.

### Plasma *N*-Glycome Analysis and MS Data Processing

*N*-Glycans were enzymatically released from plasma glycoproteins according to a previously reported protocol (28). Briefly, 5 μl of plasma from each sample was denatured by adding 10 μl of 2% SDS and incubation for 10 min at 60°C. The glycan release step was performed by the addition of 10 μl of 2.5 × PBS containing 2% Nonidet P-40 and 1 U PNGase F, followed by incubation for 16 h at 37°C. During the derivatization procedure, sialic acid residues at the nonreducing ends of the glycan were derivatized to stable end-products (α2,3-linked sialic acids were lactonized and α2,6-linked were ethyl-esterified), allowing mass-based differentiation of sialic-acid linkage variants. Briefly, 1 µl of the released plasma was added into 20 µl of derivatization reagent (250 mM HOBt and 250 mM EDC in ethanol) and incubated at 37°C for 60 min. Thereafter, glycans were purified by in-house developed cotton-based hydrophilic interaction liquid chromatography solid-phase extraction (HILIC-SPE) micro-tips as previously described (28, 30) and glycans were finally eluted into MQ water. The samples were analyzed by MALDI-TOF-MS as previously described with minor modification (24). Briefly, 1 µl of the eluted samples was mixed with 1 µl of matrix (5 mg/ml sDHB in 50% ACN with 1 mM NaOH) on a MALDI target plate and dried by air for 2 h. The measurement of the derivatized glycans was performed on a Bruker rapifleXtreme MALDI-TOF mass spectrometer fitted with a Smartbeam-3D laser in reflectron positive mode and commanded by the proprietary software flexControl 4.0 (Bruker Daltonics). Instrument calibration was achieved using the Bruker Peptide Calibration Standard II. The measurements were recorded in the *m/z* window of 1,000–5,000 with 5k laser shots in a random walking pattern of 100 shots per raster spot at the frequency of 5,000 Hz.

Raw MS data from all samples was processed at once using the same parameters. They were baseline-corrected with the TopHat method and smoothed with Savitzky Golay algorithm by flexAnalysis software and.xy files were exported for further processing. The.xy files were re-calibrated with the in-house developed software MassyTools (29) (version 0.1.8.1.2) using a selection of well-known high-intensity glycan signals distributed across the detected m/z range (minimum five calibrants at S/N >9, **Supplementary Table S1**). Plasma *N*-glycan profiles were obtained from all 75 TC, 25 BTN, 50 HC, 12 quality control standard samples, and five blanks, of which 161 profiles passed our quality criteria during the re-calibration (blanks and one standard sample was excluded due to low intensity). For the cohort, 131 peaks were manually assigned to glycan compositions using the GlycoPeakfinder tool of Glycoworkbench as well as previously confirmed glycan compositions (28, 31). Using the composition list, the intensities for the putative glycan structures were extracted as background-corrected area from the raw data with the software MassyTools. Further curation of the extracted data was done in Microsoft Excel. After further curation (S/N >9, ppm error <20, and QC score <25%), 96 glycan compositions out of the 131 compositions remained for quantitative analysis (**Supplementary Table S2**). At last, the sum of glycan areas per spectrum was re-scaled to 1 to evaluate relative intensities. In order to combine the effects of single glycans sharing similar structures and to study the general glycosylation features, such as the number of antennae of complex type *N*-glycans (CA), the level of bisection (B), fucosylation (F), galactosylation (G) and sialylation (S), 91 derived traits were calculated from the 96 directly detected glycan traits on the basis of their common structural features (32–34) (**Supplementary Table S3**).

### Experimental Design and Statistical Analysis

All 150 cohort samples (75 TC + 25 BTN + 50 HC) and 17 quality control samples consisting of five blanks (water) and 12 plasma standards were randomly distributed over two 96-well sample plates and prepared and analyzed as described above. After the removal of low-quality spectra during quality control steps, the cohort data consisted of 100 (75 + 25) cases and 50 controls. The calculations of derived glycan traits were performed in RStudio. Data quality of the cohort was assessed by the 12 standard plasma samples, which were randomly distributed in the two plates and calculating the average value, standard deviation (SD), and the coefficient of variance (CV) for all directly detected and derived glycan traits (**Supplementary Table S4**).

Direct and derived glycan traits were compared between subgroups (TC *vs*. BTN, TC *vs*. HC, and BTN *vs*. HC) using the nonparametric Mann–Whitney–Wilcoxon test since data was non-normally distributed. Multiple testing correction was used to adjust the significance threshold (P = 0.05/91—the number of derived glycan traits). The associations of glycosylation with lymph node metastasis (categorical variables) of TC were explored by logistic regression in RStudio. Derived glycan traits resulting in statistically significant p-values were further evaluated by receiver-operator-characteristics (ROC) test to assess their specificity and sensitivity in diagnosis and prediction using GraphPad Prism 8. The area under the curve (AUC) of ROC was used to assess the predictive accuracy of the glycan traits. In addition, predictive models were built by combining the altered derived glycan traits between cases and controls through logistic regression analysis in SPSS (version 23).

## Results

### Data Reliability

**Figure 1:**
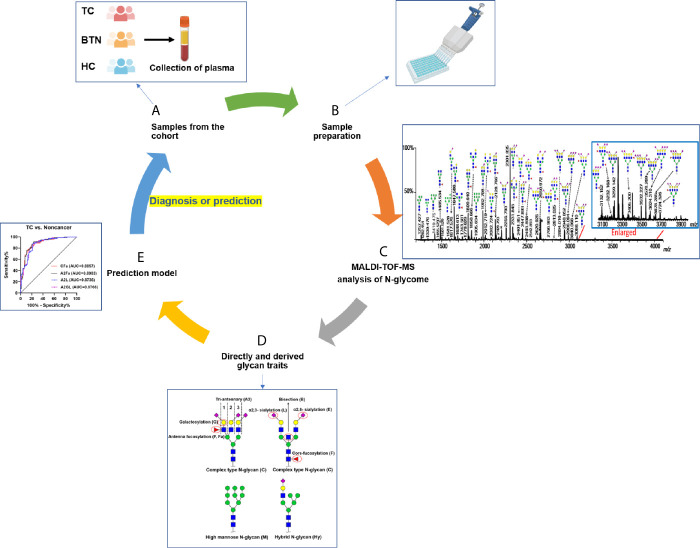
Workflow of plasma *N*-glycome analysis of TC, BTN, and HC. **(A)** Collection of plasma samples from diseases and controls; **(B)** High-throughput sample preparation including glycan release, derivatization, and enrichment; **(C)** MALDI-TOF-MS analysis of plasma *N*-glycome and data preprocessing and annotation; **(D)** Calculation of derived glycan traits; **(E)** Construction of prediction models and ROC curve analysis to test their performance. TC, thyroid cancer; BTN, benign thyroid nodules; HC, healthy controls.

The plasma *N*-glycome of patients with malignant or benign TN and matched HC (**Table 1**) were analyzed by MALDI-TOF-MS. Ninety-six of the detected glycan compositions passed our quality criteria for quantification (**Supplementary Table S2**), which were grouped into 91 derived glycan traits based on structural features of glycans including the number of antennae (CA), fucosylation (F), bisection (B), galactosylation (G), sialylation (S), and linkage-specific sialylation (**Figure 1** and **Supplementary Table S3**). As described previously, derived traits reflect the biosynthetic pathways of glycans and could facilitate interpretation of the results and biological effects (28, 32). Additionally, derived glycan traits seem to have better technical robustness compared to directly detected glycan traits (35), which was also confirmed in the present study (**Supplemental Table S4**). Average intensity, standard deviation (SD), and the relative SD (CV) from the technical replicates of 11 (one was kept out due to low quality) plasma standard samples that were randomly distributed in the plates and measured together with the cohort samples demonstrated overall method repeatability on direct- and derived-trait level (**Supplemental Table S4**). The average CV of top-20 directly detected glycan traits and all 91 derived glycan traits was 5.26 and 2.54%, respectively (**Supplemental Table S4**). Raw data files of directly detected and derived glycan traits for all the samples measured in the present study were provided (**Supplemental Table S5**).

### Identification of Plasma *N*-Glycome Alteration in TC and BTN

**Figure 2:**
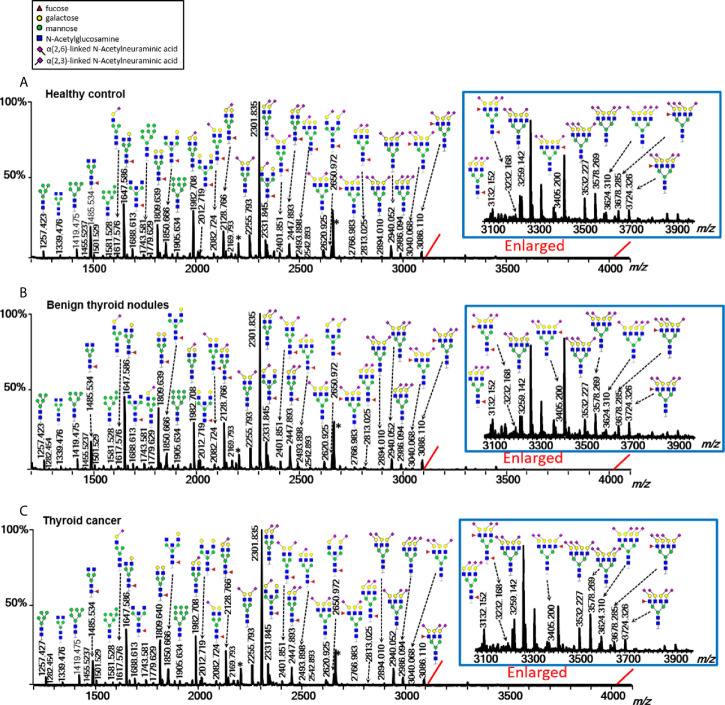
Typical MALDI-TOF-MS spectra of plasma protein *N*-glycome for **(A)** healthy control, **(B)** benign thyroid nodules, and **(C)** thyroid cancer. Spectra were recorded in positive-ion reflectron mode on a Bruker rapifleXtreme mass spectrometer. Major *N*-glycan peaks were annotated and assigned to compositions and the presence of structural isomers cannot be excluded. The asterisk (*) are by-products.

Multiple directly detected glycan traits were found differentially expressed between HC, BTN, and TC. Typical annotated MALDI-TOF-MS spectra of plasma *N*-glycomes from HC, BTN and TC were depicted in **Figure 2**, demonstrating differences in peak patterns between the three groups. As derived glycan traits have better technical robustness and could facilitate interpretation of the results and biological effects, subsequently we mainly focused on the derived glycan traits.

Plasma *N*-glycome in TC and BTN showed changes in antennarity (A) of complex type glycans compared with HC. A decrease in the antennarity was found: tetra-antennary *N*-glycans within complex type (CA4) were decreased in TC and BTN profiles than in HC samples (**Table 2** and **Supplemental Table S6**), with a concomitant increase in monoantennary *N*-glycan species (CA1; **Supplemental Table S6**). In addition, TC and BTN patients showed lower levels of fucosylation than HC, especially for poly-fucosylation (CFa and A2Fa, difucosylation), diantennary and tetra-antennary species (A2Fa, A4F, A4L0F, and A2LF; **Table 2**). In addition to fucosylation differences, TC and BTN patients displayed a higher galactosylation of tetra-antennary glycans (A4G) compared to HC (**Table 2**), which was mainly due to the increase of galactosylation of non-fucosylated tetra-antennary glycan species (A4F0G; **Table 2**). In contrast, TC and BTN had a lower galactosylation of fucosylated sialylated diantennary glycans (A2FSG) compared with HC (**Table 2**). Altered sialylation was also found in TC and BTN compared to HC. Generally, sialylation of diantennary glycans was significantly decreased and sialylation of tetra-antennary species was significantly increased in TC and BTN compared with HC (**Table 2**). For example, sialylation per antenna within tetra-antennary glycans (A4S) was higher in subjects with TC or BTN than HC (**Table 2**), which was mainly driven by the increase of sialylation of non-fucosylated tetra-antennary glycans (A4F0S; **Table 2**). Moreover, sialylation per galactose within non-fucosylated tetra-antennary glycans (A4F0GS) was higher in TC and BTN compared to HC (**Table 2**), but in fucosylated glycans (A4FGS) it was lower in TC and BTN compared with HC (**Table 2**). With regard to sialylation linkages, the changes of α2,6- and α2,3- linked sialylation within tetra-antennary species (A4E, A4L, A4F0E, A4F0L, A4FGE) were in accordance with the results of A4S, A4F0S, and A4FGS (**Table 2**). However, α2,3-linked sialylation within diantennary glycans (A2L, A2FL) showed significant decreases in TC and BTN compared to HC (**Table 2**). We did not find differences of bisection (B) between TN (TC + BTN) and HC (**Supplemental Table S6**).

**Table 2:** Differentially expressed derived glycan traits contributing to distinguishing between groups of patients with thyroid diseases and healthy controls.

Glycan traits	Descriptions	Median of TC	Median of BTN	Median of HC	P-value		
					TC *vs.* BTN	TC *vs.* HC	BTN *vs.* HC
	**General**						
**CA4**	tetraantennary species (A4) in complex glycans	0.0274	0.0267	0.0329	0.2217	**2.47E−12**	**3.35E−07**
	**Fucosylation (F)**						
**A4F**	in tetraantennary (A4)	0.3266	0.3164	0.4349	0.4911	**1.20E−12**	**1.48E−09**
**A2LF**	in diantennary (A2) with α2,3-sialylation	0.5864	0.6050	0.6525	0.2500	**2.22E−15**	**3.10E−06**
**A4L0F**	in tetraantennary (A4) without α2,3-sialylation	0.4688	0.4090	0.5692	0.0461	**5.69E−09**	**2.35E−06**
**CFa**	antenna-fucosylation in complex glycans	0.0339	0.0393	0.0464	**6.12E−05**	**1.31E−22**	**1.00E−05**
**A2Fa**	antenna-fucosylation in diantennary (A2)	0.0393	0.0446	0.0538	**8.00E−05**	**3.37E−24**	**2.93E−06**
	**Galactosylation(G)**						
**A4G**	in tetraantennary (A4)	0.8238	0.8530	0.7383	0.2248	**5.73E−14**	**2.09E−09**
**A2FSG**	in fucosylated sialylated diantennary (A2)	0.9682	0.9683	0.9713	0.6702	**5.08E−05**	6.88E**−**04
**A4F0G**	in non-fucosylated tetraantennary (A4)	0.6734	0.6836	0.5651	0.4911	**1.20E−12**	**1.48E−09**
	**Sialylation (S)**						
**A4S**	in tetraantennary (A4)	0.6162	0.6225	0.5305	0.8391	**2.89E−15**	**1.20E−09**
**A4F0S**	in non-fucosylated tetraantennary (A4)	0.4663	0.4647	0.3670	0.8020	**1.71E−14**	**4.44E−09**
**A4FGS**	per galactose in fucosylated tetraantennary (A4)	0.9527	0.9518	0.9638	0.2843	**2.42E−09**	**1.74E−04**
**A4F0GS**	per galactose in non-fucosylated tetraantennary (A4)	0.7029	0.6942	0.6397	0.0714	**7.19E−12**	**3.83E−04**
	**α2,3-sialylation (L)**						
**A2L**	in diantennary (A2)	0.0798	0.0879	0.0962	**5.53E−05**	**8.88E−16**	**1.59E−04**
**A2FL**	in fucosylated diantennary (A2)	0.1216	0.1331	0.1605	0.0040	**4.22E−14**	**6.93E−05**
**A4L**	in tetraantennary (A4)	0.2498	0.2538	0.1912	0.7351	**7.79E−14**	**4.15E−09**
**A4F0L**	in non-fucosylated tetraantennary (A4)	0.1955	0.1891	0.1419	0.7959	**2.44E−13**	**7.78E−08**
**A2GL**	per galactose in diantennary (A2)	0.0891	0.0984	0.1052	**1.95E−05**	**1.55E−15**	**5.15E−04**
**A4FGL**	per galactose in fucosylated tetraantennary (A4)	0.3545	0.3976	0.2826	0.0619	**1.69E−07**	**1.59E−05**
	**α2,6-sialylation (E)**						
**A4E**	in tetraantennary (A4)	0.3668	0.3634	0.3390	0.2989	**5.11E−15**	**6.86E−08**
**A4FE**	in fucosylated tetraantennary (A4)	0.0910	0.0914	0.1191	0.6936	**4.20E−11**	**7.12E−09**
**A4F0E**	in non-fucosylated tetraantennary (A4)	0.2773	0.2734	0.2245	0.7232	**5.53E−14**	**6.23E−09**
**A4FGE**	per galactose in fucosylated tetraantennary (A4)	0.5967	0.5510	0.6807	0.0631	**5.55E−08**	**1.59E−05**

Descriptions of the derived traits, median values of derived glycan traits in TC, BTN, and HC as well as p-values for the comparison by Mann–Whitney U test for the cohort are shown. The p values considered significant are below the significance threshold of 5.49E−4 (= p-value of 0.05 after multiple testing correction for 91 derived traits). The p-values highlighted indicated significance. Red and blue indicate the direction of changes up-regulation and down-regulation, respectively. Derived traits in gray shading showed the potential of distinguishing among the three groups of TC, BTN, and HC. TC, thyroid cancer; BTN, benign thyroid nodules; HC, healthy control. The subject of the derived traits calculation is represented by the last letter, e.g., galactosylation (G), and the group on which it is calculated by the preceding letters, e.g., fucosylated sialylated diantennary species (A2FS). This, for instance, translates A2FSG into the galactosylation per antenna within fucosylated sialylated diantennary glycans.

**Figure 3:**
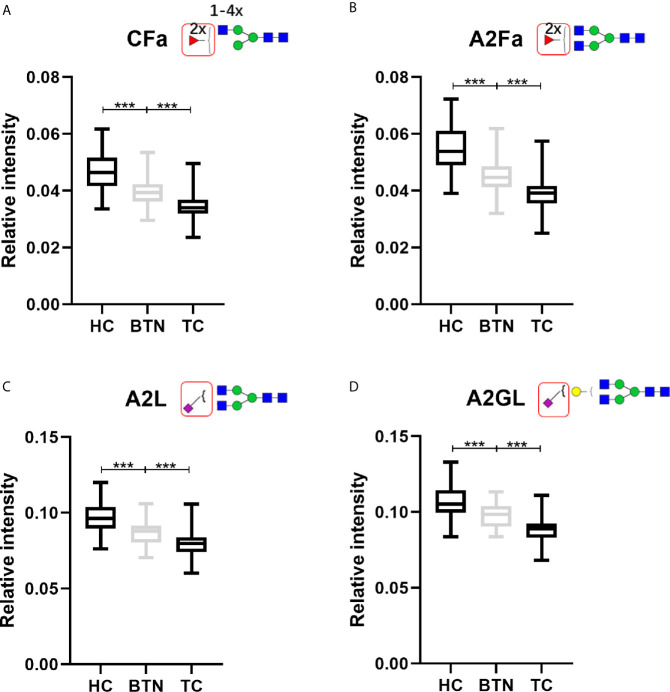
The boxplots of derived glycan traits **(A)** CFa, **(B)** A2Fa, **(C)** A2L, and **(D)** A2GL, which showed consecutive changes in BTN and TC compared to HC. Numerical values indicate compositional limitations (i.e. 1-4 as the possible number of N-acetylhexosamines). Please see **Supplemental Table S3** for more detailed derived glycan trait descriptions. Glycan trait abbreviations: C, within complex; Fa, species with 2 fucoses (i.e. at least one antennary fucose); A2, diantennary; L, α2,3-linked sialylation; G, galactose. *** represents p-value < 0.001 (after multiple testing correction).

Interestingly, BTN and TC showed very similar patterns of plasma glycans (**Table 2**). Most of the altered derived glycan traits mentioned above showed no difference between TC and BTN (**Table 2**), which means these traits were associated with TN (BTN + TC) and could be used to distinguish TN and HC, but could not differentiate between benign and malignant TN. Notably, four of these altered derived glycan traits, namely CFa, A2Fa, A2L, and A2GL, were significantly different among the three subgroups of TC, BTN, and HC (**Table 2** and **Figure 3**). Moreover, these four traits showed consecutive decreases from HC to BTN, and from BTN to TC (**Table 2** and **Figure 3**). This indicated that the four derived glycan traits might have potential as biomarkers for differential diagnosis of benign and malignant TN, as further investigated below.

### Associations of Plasma *N*-Glycome With Lymph Node Metastasis in TC

The associations of plasma *N*-glycome with lymph node metastasis in TC were explored by logistic regression, for which only derived glycan traits that showed differences between TC patients with and without lymph node metastasis were included (**Supplemental Table S7**). Fucosylation within diantennary glycans with α2,3-linked sialic acid (A2LF) was found to be significantly positively associated with lymph node metastasis in TC (P = 0.003826; **Supplemental Table S7**). In contrast, α2,3-sialylation within non-fucosylated di- or tri-antennary glycans (A3F0L, A2F0L, and A2F0GL) was strongly negatively associated with lymph node metastasis (P <0.01; **Supplemental Table S7**).

### Performance of Plasma Glycan Traits in Identifying TC and BTN

**Figure 4:**
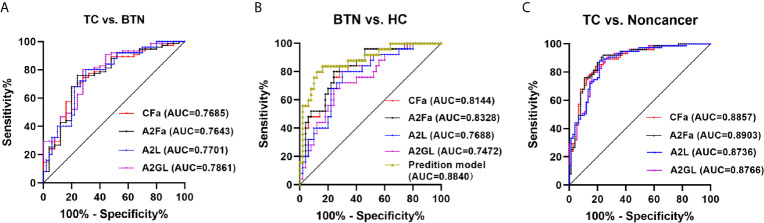
ROC curve analysis for differential expressed derived glycan traits and prediction model based on them. **(A)** TC *vs*. BTN, **(B)** BTN *vs*. HC, **(C)** TC *vs*. Noncancer (BTN + HC). TC, thyroid cancer; BTN, benign thyroid nodules; HC, healthy controls.

ROC curves were assessed for the selected four derived glycan traits. The resulting ROC curve demonstrated the potential of CFa, A2Fa, A2L, and A2GL in identifying benign and malignant TN (**Figure 4**). According to our results, the AUCs of CFa, A2Fa, A2L, and A2GL were 0.7685, 0.7643, 0.7701, and 0.7861 when discriminating between malignant TN and benign TN (**Figure 4A**). Moreover, the AUCs of the four traits were 0.8144, 0.8328, 0.7688, and 0.7472 in differentiating BTN from HC (**Figure 4B**). Furthermore, the performance of the four traits was good with AUCs of 0.8857, 0.8903, 0.8736, and 0.8766 in the differential diagnosis of patients with TC and noncancer (BTN + HC) (**Figure 4C**). Finally, predictive models were built by logistic regression analysis in SPSS (version 23). Initially, the four derived traits of CFa, A2Fa, A2L, and A2GL were used for the models. Multiple combinations of these four traits were then evaluated with regard to predictive accuracy, resulting in the final models: For distinction of TC and BTN, the prediction model only included A2GL. For the distinction of BTN and HC, the prediction model was composed of CFa, A2Fa, and A2GL. For the distinction of TC and Noncancers, the prediction model only included A2Fa (**Figure 4**). Our results suggested that the prediction models including least number of traits achieved best performance in the differential diagnosis (**Figure 4**).

### Performance of Plasma Glycan Traits in Stratifying TC to Low or High Risk of Lymph Node Metastasis

**Figure 5:**
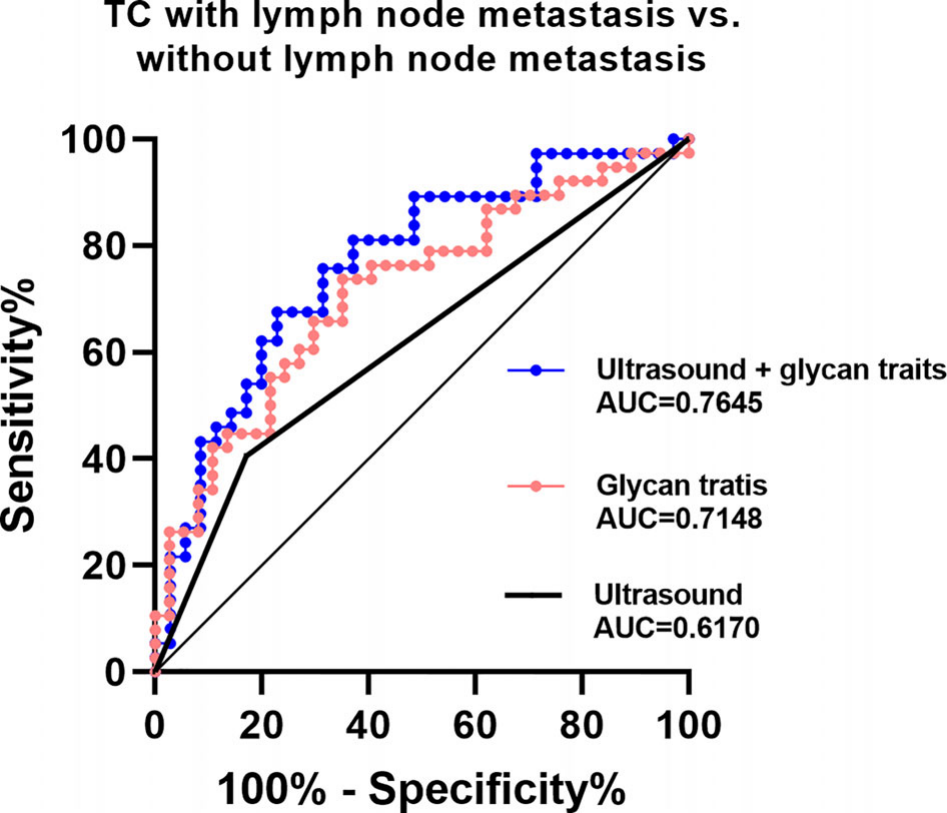
ROC curve analysis for derived glycan traits which showed associations with lymph node metastasis.

The existing method for predicting lymph node metastasis is ultrasound. Considering that A2LF, A3F0L, A2F0L, and A2F0GL showed strong associations with lymph node metastasis in TC, we attempted to build predictive models for identifying lymph node metastatic thyroid cancer based on these glycan traits. The performance of the established prediction models in predicting TC with or without lymph node metastasis was evaluated by ROC curves (**Figure 5**). Our results showed that the AUC value of ultrasound in predicting metastasis was 0.6170 (**Figure 5**), suggesting an “uninformative” test. The AUC value of the prediction model consisted of A2LF, A3F0L, A2F0L, and A2F0GL was 0.7148 (**Figure 5**), suggesting a “moderately accurate” diagnostic test. The performance was further improved when combing the four glycan traits with ultrasound with an AUC of 0.7645 (**Figure 5**).

## Discussion

TN are the most common thyroid disease and 10% of them are with a high risk of TC. Furthermore, TC is the most common endocrine tumor. Although the mortality rate has remained stable, the incidence rate of the TC has increased substantially (36). Deeper insight into the pathophysiology and screening of diagnostic and prognostic biomarkers is crucial for TC. Profiling of protein *N*-glycosylation with functional impact on the proteins has a high potential for advancing this. So far, aberrant glycosylation in TC has been reported in limited studies which mainly focused on glycosylation changes in cells, tissues, and plasma IgG (25–27). For example, employing MALDI-TOF(/TOF)-MS, researchers found four sialylated *N*-glycans and two high-mannose type *N*-glycans were significantly different between formalin-fixed paraffin-embedded tissues of PTC and adjacent normal tissues (26). In addition, Chen et al. detected aberrant serum IgG Fc glycosylation profiles in TC (27). Nevertheless, little is known regarding total plasma glycosylation changes in TC and BTN. Zhang et al. developed an integrated method for comprehensive *N*-glycoproteome profiling of human biofluids (37). By this method, desialo-*N*-glycopeptides from the urine and plasma of HC, PTC, and PTC with Hashimoto’s thyroiditis were analyzed. Finally, they identified 92 altered proteins and 134 intact *N*-glycopeptides from the plasma and urine samples of the three groups and revealed a novel indicator (ratio of fucosylated to nonfucosylated *N*-glycopeptide) contributing to clinical TC diagnostics (38). The published study by Zhang et al. mainly focused on glycopeptides (glycoproteomics) to obtain the glycosylation information in TC and controls and removed the sialic acid residues at the ends of the glycans when doing the analysis. In addition, the exiting study did not include BTN. In contrast, the present study focused on released glycans (glycomics) from the plasma of HC, BTN, and TC, including the linkage-specific sialic acids information.

The present study represents the first comprehensive analysis of the plasma *N*-glycome in TC and BTN. Importantly, several glycosylation features were found for the first time to differ between BTN, TC, and HC, namely complexity, galactosylation, fucosylation, and sialylation. Especially, with regard to sialylation, our approach included the discrimination of functionally disparate α2,3- and α2,6- linkages types. In addition, we found consecutive decreases of CFa (difucosylation), A2Fa (difucosylation), A2L (α2,3-sialylation in A2), and A2GL (α2,3-sialylation per galactose in A2) in BTN and TC compared with HC. These four derived glycan traits and prediction models based on them showed relatively good performance with “moderately accurate” to “accurate” AUC values, suggesting plasma *N*-glycome patterns may have potential as novel biomarkers for identifying TC and BTN assisting the existed diagnostic methods (such as ultrasound and FNA). Nevertheless, the sample size of BTN is not large enough in the present study. Moreover, testing, training, and validation samples are always needed during the discovery of cancer biomarkers (39). The results we obtained in the present study still need independent validation in large cohorts, which is one of the limitations of this study. Interestingly, though many glycan traits were changed in BTN and TC compared to HC, BTN is very similar to TC in plasma *N*-glycome patterns (**Table 2**), which reminds us including benign diseases as disease control is very important during the discovery of cancer biomarkers.

Our investigation of dysregulation of *N*-glycan patterns in TC may point at pathophysiological processes involving multiple proteins, as we discuss below. Fucosylation is one important mode of glycosylation in TC and is regulated by several kinds of fucosyltransferases. FUT3, 4, and 6 are responsible for antennary fucosylation (leading to the multiple fucoses). FUT4 was identified as an independent marker for PTC (40). In other types of cancer, FUT3 was involved in the proliferation, migration, tumorigenesis of pancreatic cancer cells (41). While, FUT5 and FUT6 were reported to be associated with the development of colorectal cancer (42). These results may help to explain part of the possible mechanism of dysregulated CFa (difucosylation) and A2Fa (difucosylation) in BTN and TC in this study. Nevertheless, the glycan traits containing fucosylation in the present study differ from what has been reported in other cancers, such as increased serum fucosylation (A2LF, A3LF, and A4LF) was observed in pancreatic cancer (43) and A3Fa were found increased in colorectal cancer (15). This indicated that alterations of plasma protein fucosylation might be cancer-specific, making plasma *N*-glycome patterns more promising as potential cancer-specific biomarkers. In addition, sialic acids are directly involved in the activation and modulation of the immune system, which depends on the linkage (44, 45). Our novel method enabled us to discriminate between the two types of linkage (α2,3 or α2,6) and get linkage-specific data of sialylation. We found that α2,3-sialylation within A2 was consecutively decreased in BTN and TC. These glycans may come from liver-produced acute phase proteins (32). Consistently, proteomic analysis revealed decreased levels of liver-derived glycoproteins (such as apolipoprotein A4, apolipoprotein C-I, apolipoprotein C-III, and alpha-1 antitrypsin) in PTC compared to BTN or HC (46–49). Besides, Arcinas et al. profiled secreted and cell surface glycoproteins of thyroid cancer cells using a glyco-capture method. Among the 397 proteins identified within the PTC cell line (TPC-1), 37 were identified as secreted glycoproteins, which may also contribute to the changed levels of *N*-glycans in the plasma of TC patients (50). Increased α2,3-linked sialylation in plasma has been supposed to be involved in the anti-inflammatory effects and has been reported in diseases such as IBD (51). Reduced α2,3-linked sialylation in TC, which is opposite to the status in other diseases, might reflect other processes that are not related to anti-inflammation and classical immune response. For example, hormones may be involved in the regulation of glycosylation in TC (26, 52). Since data on linkage-specific sialylation in diseases is scarce, the exact mechanisms of reduced α2,3-linked sialylation in TC need to be further studied. What’s more, α2,6-sialylated glycans (H5N4E1, H5N4F1E1, H5N4F1L1E1, and H5N4F1E2) were found to increase in tissues of PTC (26), which were consistent with our results in plasma (**Table 2**). However, we did not find α2,6-sialyation differences between TC and BTN (**Table 2**).

In this study, we observed that A2LF, A3F0L, A2F0L, and A2F0GL were significantly associated with lymph node metastasis in TC and models constructed from the four glycan traits have high potential as predictive biomarkers. The combination of fucosylation with α 2,3-linked sialylation (LF) often suggests the terminal sialyl-Lewis X epitopes (32). The associations between sialyl-Lewis X on liver-derived proteins and metastasis have been reported in many types of cancer, such as breast cancer (53, 54), liver cancer (55), and renal cancer (56). Interestingly, the proteomic analysis showed that levels of liver-produced glycoproteins such as alpha-1-antitrypsin, which may be the origin of the glycan traits mentioned above, were associated with invasion and metastasis in PTC (57). Additionally, epithelial to mesenchymal transition (EMT), which is a key step in the metastatic process of cancer, is triggered by the secreted cytokine TGF-β (58), while fucosylation is important for the functions of TGFβ-R (25). The novel link of the lymph node metastasis of TC with fucosylation (A2LF) and α2,3-sialylation (A3F0L, A2F0L, and A2F0GL) was for the first time indicated in the present study, providing potential glycan biomarkers to stratify TC into low or high risk of lymph node metastasis.

The methodology used in the present study doesn’t provide detailed information on the plasma protein origin of the glycan biomarkers. This limitation is well known in the field of glycobiology and can be addressed by protein-specific glycomic (glycoproteomic) analyses. However, it is still a big challenge in terms of sensitivity, throughput, and discrimination of glycan linkage information. In addition, measurement of thyroglobulin in washout fluid increases specificity and sensitivity of lymph node metastasis. However, the data for the thyroglobulin in washout fluid is not available for us. The results for the comparison between ultrasound and the *N*-glycan traits in the present study may not be comprehensive. In future studies, measurement of thyroglobulin in washout fluid from the needle used for lymph node aspiration cytology should be considered. On the other hand, studies in large validation cohorts and prospective investigations are still needed to validate our findings before the application of the biomarkers.

## Conclusion

To our knowledge, this is the first study to identify plasma *N*-glycome in TC and BTN to date and included novel linkage-specific sialylation information. Plasma glycosylation was proven to differ between BTN, TC and HC in main glycosylation features. We also revealed unreported associations between plasma glycan features and lymph node metastasis of TC. Several derived glycan traits and prediction models based on them showed high potential as biomarkers for differential diagnosis of BTN and TC and stratifying TC patients, which can function as a base for the development of blood-based tests. Future studies, preferably in a longitudinal and protein-specific manner, are warranted to assess the potential for early detection and surveillance based on the here reported plasma *N*-glycan features. Moreover, genetic studies including the expression of glycosyltransferases and glycosidases should improve insight into the mechanisms involved. Overall, this study enhanced the understanding of TC.

## Data Availability Statement

The original contributions presented in the study are included in the article/**Supplementary Material**. Further inquiries can be directed to the corresponding author.

## Ethics Statement

The studies involving human participants were reviewed and approved by the regional ethics committee of the Peking Union Medical College Hospital. The patients/participants provided their written informed consent to participate in this study.

## Author Contributions

XX and ZZ conceived and initiated this study. ZZ performed the experiments and data analysis and interpreted the results with support of KR. ZZ, ZL and JW collected samples and clinical parameters. ZZ prepared the figures and tables, and wrote the original draft with support from KR and XX. All authors contributed to the article and approved the submitted version.

## Funding

The work was supported by National Natural Science Foundation of China (32071436, 31901041).

## Conflict of Interest

The authors declare that the research was conducted in the absence of any commercial or financial relationships that could be construed as a potential conflict of interest.

## Abbreviations

AUC, area under the curve; BTN, benign thyroid nodules; CV, coefficient of variance; EMT, epithelial-to-mesenchymal transition; FNA, fine-needle aspiration; HC, healthy controls; HILIC-SPE, hydrophilic interaction liquid chromatography solid-phase extraction; ITN, indeterminate thyroid nodules; MALDI-TOF-MS, matrix-assisted laser desorption/ionization time-of-flight mass spectrometry; PTC, papillary thyroid carcinoma; ROC, receiver-operator-characteristics; SD, standard deviation; TC, thyroid cancer; TN, thyroid nodules.
