# Supplementary material for: Distinguishing Benign and Malignant Thyroid Nodules and Identifying Lymph Node Metastasis in Papillary Thyroid Cancer by Plasma N-Glycomics
Source: Front Endocrinol (Lausanne). 2021 Jun 25;12:692910. doi: 10.3389/fendo.2021.692910 (PMC8267918; doi:10.3389/fendo.2021.692910)
Supplement: Supplementary file 1 [file DataSheet_1.pdf]

Supplementary materials

Distinguishing benign and malignant thyroid nodules and identifying lymph node metastasis in papillary thyroid cancer by plasma *N*-glycomics

Zejian Zhang[1], Karli R. Reiding[2,3], Jianqiang Wu[1], Zepeng Li[4], Xiequn Xu[5*]

[1]Department of Medical Research Center, State Key Laboratory of Complex Severe and Rare Diseases, Peking Union Medical College Hospital, Chinese Academy of Medical Sciences and Peking Union Medical College, Beijing, China
[2]Biomolecular Mass Spectrometry and Proteomics, Bijvoet Center for Biomolecular Research and Utrecht Institute for Pharmaceutical Sciences, University of Utrecht, Utrecht, the Netherlands
[3]Netherlands Proteomics Center, Utrecht, the Netherlands
[4]Department of Clinical Laboratory, Peking Union Medical College Hospital, Peking Union Medical College and Chinese Academy of Medical Sciences
[5]Department of General Surgery, Peking Union Medical College Hospital, Chinese Academy of Medical Sciences and Peking Union Medical College, Beijing, China

* Correspondence:
Xiequn Xu
xxq75@163.com

Detailed protocols for plasma *N*-glycome analysis

Materials

Sodium dodecyl sulfate (SDS), trifluoroacetic acid (TFA), HPLC-grade ACN and ethanol were purchased from Merck. Disodium hydrogen phosphate dihydrate ($Na_2HPO_4 \times 2\ H_2O$), potassium dihydrogenphosphate (KH2PO4), sodium chloride (NaCl), Nonidet P-40 substitute (NP-40), 1-hydroxybenzotriazole monohydrate (HOBt) and super-2,5-dihydroxybenzoic acid (DHB) were obtained from Sigma Aldrich. 1-ethyl-3-(3-(dimethylamino)propyl)-carbodiimide (EDC) was obtained from Fluorochem. Peptide-N-glycosidase F (PNGase F) was purchased from Roche Diagnostics and calibration peptide mix for MALDI-TOF-MS measurements was obtained from Bruker Daltonics. Ultrapure water (MQ) used in the present study was obtained from Waters.

Plasma *N*-glycan release

N-Glycans were enzymatically released from the human plasma glycoproteins according to a previously reported method by Karli R. Reiding *et al*[1]. Briefly, 5 μL of plasma from each sample was denatured by adding 10 μL of 2% SDS and incubation for 10 min at 60 °C. The glycan release step was performed by the addition of 10 μL of 2.5 × PBS containing 2% Nonidet P-40 and 1 U PNGase F, followed by incubation for 16 h at 37 °C.

Derivatization and purification for released *N*-glycans

During the derivatization procedure, sialic acid residues at the nonreducing ends of the glycan were derivatized to stable end-products (α2,3-linked sialic acids were lactonized and α2,6-linked were ethyl-esterified), allowing mass-based differentiation of sialic-acid linkage variants[1]. Briefly, 1 μL of the released plasma was added into 20 μL of derivatization reagent (250 mM HOBt and 250 mM EDC in ethanol) and incubated at 37°C for 60 min, followed by adding 20 μL of ACN and 15 min further incubation at −20 °C before the glycan purification.

Released *N*-glycans were purified using our in-house developed cotton-based hydrophilic interaction liquid chromatography solid-phase extraction (HILIC-SPE) micro-tips as previously reported[1,2]. In short, the cotton tips were pre-conditioned with 3 × 20 μL of MQ water, followed by equilibration with 3 × 20 μL of 85% ACN. Then, the sample was loaded by pipetting the derivatized glycan mixture 20 × extensively, followed by the washing steps consisting of 3 × 20 μL of 85% ACN + 1% TFA and 3 × 20 μL of 85% ACN. Glycans were finally eluted into 10 μL of MQ water.

MALDI-TOF-MS detection of the purified *N*-glycans

The samples were analyzed by MALDI-TOF-MS as previously described with minor modification[2,3]. Briefly, 1 μL of the eluted samples was mixed with 1 μL of the matrix (5 mg/mL sDHB in 50% ACN with 1 mM NaOH) on a MALDI target plate and dried by air for two hours. The measurement of the derivatized glycans was performed on a

Bruker rapifleXtreme MALDI-TOF mass spectrometer fitted with a Smartbeam-3D laser in reflectron positive mode and commanded by the proprietary software flexControl 4.0 (Bruker Daltonics). Instrument calibration was achieved using the Bruker Peptide Calibration Standard II. The measurements were recorded in the *m/z* window of 1000-5000 with 5k laser shots in a random walking pattern of 100 shots per raster spot at the frequency of 5000 Hz.

MS data processing

Raw MS data from all samples was processed at once using the same parameters. They were baseline-corrected with the TopHat method and smoothed with Savitzky Golay algorithm by flexAnalysis software, and .xy files were exported for further processing. The .xy files were re-calibrated with the in-house developed software MassyTools[4] (version 0.1.8.1.2) using a selection of well-known high-intensity glycan signals distributed across the detected *m/z* range (minimum five calibrants at S/N > 9, Supplementary Table S1). Plasma *N*-glycan profiles were obtained from all 75 TC, 25 BTN, 50 HC, 12 quality control standard samples, and 5 blanks, of which 161 profiles passed our quality criteria during the re-calibration (blanks and one standard sample was excluded due to low intensity). For the cohort, 131 peaks were manually assigned to glycan compositions using the GlycoPeakfinder tool of Glycoworkbench [5] as well as previously confirmed glycan compositions[1,3] and a composition list for the targeted data extraction was generated. Using the composition list, the intensities for the putative glycan structures were extracted as background-corrected area from the raw data with the software MassyTools. Further curation of the extracted data was done in Microsoft Excel. After further curation (S/N > 9, ppm error < 20, and QC score <25%)[3], 96 glycan compositions out of the 131 compositions remained for quantitative analysis (Supplementary Table S2). At last, the sum of glycan areas per spectrum was re-scaled to 1 to evaluate relative intensities. In order to combine the effects of single glycans sharing similar structures and to study the general glycosylation features, such as the number of antennae of complex-type *N*-glycans (CA), the level of bisection (B), fucosylation (F), galactosylation (G) and sialylation (S), 91 derived traits were calculated from the 96 directly detected glycan traits based on their common structural features [1,3,6] (Supplementary Table S3).

Statistical analysis

Direct and derived glycan traits were compared between subgroups (TC vs. BTN, TC vs. HC, and BTN vs. HC) using the nonparametric Mann-Whitney-Wilcoxon test since data was non-normally distributed. Multiple testing correction was used to adjust the significance threshold (P=0.05/91--the number of derived glycan traits). The associations of glycosylation with lymph node metastasis (categorical variables) of TC were explored by logistic regression in RStudio. Derived glycan traits resulting in statistically significant p-values were further evaluated by receiver-operator-characteristics (ROC) test to assess their specificity and sensitivity in diagnosis and prediction using GraphPad Prism 8. The area under the curve (AUC) of ROC was used to assess the predictive accuracy of the glycan traits. If the AUC was greater than 0.9,

the tests were considered to be "highly accurate," while between 0.8 and 0.9 indicated "accurate." When the AUC was between 0.7 and 0.8, the test was deemed "moderately accurate." In addition, predictive models were built by combining the altered derived glycan traits between cases and controls through logistic regression analysis in SPSS (version 23).

References

1. Reiding KR, Blank D, Kuijper DM, Deelder AM, Wuhrer M. High-throughput profiling of protein N-glycosylation by MALDI-TOF-MS employing linkage-specific sialic acid esterification. *Anal Chem* 2014; **86**(12): 5784-93.
2. Selman MH, Hemayatkar M, Deelder AM, Wuhrer M. Cotton HILIC SPE microtips for microscale purification and enrichment of glycans and glycopeptides. *Anal Chem* 2011; **83**(7): 2492-9.
3. Zhang Z, Westhrin M, Bondt A, Wuhrer M, Standal T, Holst S. Serum protein N-glycosylation changes in multiple myeloma. *Biochim Biophys Acta Gen Subj* 2019; **1863**(5): 960-70.
4. Jansen BC, Reiding KR, Bondt A, et al. MassyTools: A High-Throughput Targeted Data Processing Tool for Relative Quantitation and Quality Control Developed for Glycomic and Glycoproteomic MALDI-MS. *J Proteome Res* 2015; **14**(12): 5088-98.
5. Ceroni A, Maass K, Geyer H, Geyer R, Dell A, Haslam SM. GlycoWorkbench: a tool for the computer-assisted annotation of mass spectra of glycans. *J Proteome Res* 2008; **7**(4): 1650-9.
6. Clerc F, Reiding KR, Jansen BC, Kammeijer GS, Bondt A, Wuhrer M. Human plasma protein N-glycosylation. *Glycoconj J* 2016; **33**(3): 309-43.
